# *The Organon* and Materia Medica Club of the Bay Cities of California

**Published:** 1896-06

**Authors:** W. E. Ledyard

**Affiliations:** Secretary


### *THE ORGANON* AND MATERIA MEDICA CLUB OF THE BAY CITIES OF CALIFORNIA.

The regular semi-monthly meeting was held at the office of Dr. George H. Martin, 606 Sutter St., San Francisco, Friday evening, January 3d, 1896.

Members present: Drs. J. M. Selfridge, W. E. Ledyard, N. T. Wilson, G. J. Augur, S. E. Chapman, George H. Martin, and C. M. Selfridge. Visitors: Drs. A. J. Forget, C. J. Holmgren, and Mr. A. Johnson.

The meeting was called to order at 8.10 o'clock by the President, Dr. J. M. Selfridge. The minutes of the previous meeting were read by the Secretary and approved.

Upon motion of Dr. Wilson, seconded by Dr. Ledyard, the courtesies of the Club were extended to Dr. Holmgren.

Dr. Chapman stated that in the case which he presented to the Club at its last meeting, upon further investigation he had discovered the patient has been suffering from constipation for a long time; the bowels retracted, with *sensation as if the bowels were drawn back to the spine*, and sheep-dung stool. He prescribed *Plumbum* $^{200}$, and thought now that on the whole the man is mending.

Dr. Ledyard thought that physicians often make the mistake of treating diarrhœa when it appears in certain cases, instead of leaving it alone and prescribing for the other symptoms.

The President then appointed Dr. Martin reader of the evening, who commenced *The Organon* at Section 29.

### Discussion.

Dr. Wilson—I presume what is meant by "life-long diseases" is that they are inherited diseases.

Dr. Ledyard—This would show the utter uselessness of resorting to hydropathy or massage to cure these chronic diseases.

Dr. Chapman—I would like to know if there is anything in our law to prohibit a man from using hydropathy? I saved two lives by this method. One case of pneumonia in a woman sixty-seven years of age. Respiration was above 50; pulse so fast that I could not count it at all; temperature 107°–108°. She was going down fast, and I saw that something more should be done. *I wrapped her in wet, cold sheets,* and the steam fairly rolled from her. A copious watery discharge from the bowels came on and relieved the tension she was under. Was this justifiable for a homœopath to do? I know that the cold water set about a reaction with this critical discharge.

Dr. J. M. Selfridge—You were not certain that she would not have had that critical discharge anyway.

Dr. Chapman—The other case was one of typhoid fever in a powerful young fellow. Rigors every evening about 10 o'clock, with tremendous shaking for hours. I could not stop it and thought he would die. I gave the indicated remedy as far as I could get at it. The rigors continued all night long. The third night I made up my mind that something more had to be done. The temperature was very high. I ordered some *hot water,* dipped some blankets in it and rolled him up in them and he slept nearly all night. He was relieved in about ten minutes after being rolled in the blankets. I continued giving the remedies. I cannot help believing that the hot water pulled the man through. I think it a very valuable auxiliary.

Dr. J. M. Selfridge—It was not Homœopathy anyway.

Dr. C. M. Selfridge—The old school are doing this a great deal in typhoid fever. They dip the patient in the water for from three to five minutes, and do this two or three times a day.

Dr. Wilson—Why would it be objectionable to Homœopathy? It simply brings about a reaction and the medicines are set to work.

Dr. J. M. Selfridge—Because it is not in accordance with the law of similars.

Dr. Chapman—The man in my case was not chilly, but simply had rigors; perhaps they were nervous.

Dr. Ledyard—It *was* homœopathic; the man was hot with fever and the water was hot.

Dr. J. M. Selfridge—Would hot water produce these symptoms?

Dr. Ledyard—It is on *the same principle as using snow for frost-bite,* and *I* call that homœopathic.

Dr. Chapman—Was I right in doing what I did?

Dr. Ledyard—Most decidedly.

Dr. J. M. Selfridge—You can apply cold, but it is a question whether or not you will be applying heat after all by using the snow in frost-bite.

Dr. Martin—In applying the snow you simply prevent the heat from coming on too suddenly. It is a mechanical condition. Persons working in a high temperature do not jump right in at once, but gradually get accustomed to it. So it is with this: we use the snow to prevent the blood from going into the frost-bitten part too suddenly.

Dr. Ledyard—It is because of the harmony of the law. What you say is true of course, but *it is in harmony with the law.*

Dr. Wilson—Simply a mechanical means then brought this condition to an end.

Dr. Martin—I believe the remedy had a great deal to do with the watery discharge from the bowels in Dr. Chapman's case. Cold applications where the temperature is very high do reduce the temperature, but this is an extremely dangerous procedure. They say that a patient with such a high temperature cannot catch cold. This may be true, but it is a tremendous shock to the system, and it is a question whether the patient will survive it or not. The after-results are sometimes very disastrous.

Dr. C. M. Selfridge—The old-school men claim that they save a great many cases by this method.

Dr. Martin—They do not save cases that we would save. When they use the cold-water treatment, *they do not use remedies.*

Dr. J. M. Selfridge—That is why they save them.

Dr. Augur—When I was practicing allopathy I used to put a person in warm water and then add cold water gradually. I had very good results from this treatment, but do not do it now.

Dr. J. M. Selfridge—Dr. Chapman's wet, hot sheet is not homœopathic, and in using it it is done like the allopaths to-day use poultices.

Dr. Chapman—*Anything* that I can do that will add to the patient's comfort, *that will not interfere with the action of the indicated remedy*, I feel that I can do.

Dr. J. M. Selfridge—Some of the high potency men discourage even douches, etc., because they say it takes away the symptoms for the indicated remedy. How do you know that it does not interfere with the indicated remedy?

Sections 30 and 31 were read.

### Discussion.

Dr. J. M. Selfridge—Hahnemann inculcates the idea that when our systems are in a healthy state, diseases will have no effect.

Dr. Martin—I would suppose that that would have reference to malaria, etc.; anything from noxious gases.

Dr. Chapman—In the country I have seen a sporadic case of diphtheria, away above civilization line, with environments perfectly sanitary, and yet had a most terrible case. It makes me a little dubious about the germ theory. I have also seen typhoid fever in a camp where it would seem impossible.

Dr. J. M. Selfridge—The causes around camps may be quite sufficient to produce typhoid.

Sections 32 and 33 were read.

### Discussion.

Dr. Chapman—These sections give me a new thought. It is a blessed good thing that *we are more susceptible to drug force than to disease,* otherwise we would have no control whatever in disease.

Dr. Martin—It may take a stronger dose to affect some persons more than others. Some persons are not affected by doses of noxious gases.

Dr. J. M. Selfridge—It is on the same principle as commencing by taking little by little until you can take in one dose what would kill twenty men. I knew of a man with disease of the mesenteric glands who took five grains of Morphine twice a day.

Dr. Martin—I had a case in which one dram of Morphine was taken every day.

Dr. Augur—I had a case in which the same thing was done.

Dr. Ledyard—For how long was it taken?

Dr. Augur—Until the patient died.

Dr. J. M. Selfridge—In regard to what Hahnemann says about *Belladonna* being a prophylactic. *It is an antidote to a certain form of scarlet fever* but not to others.

Dr. Wilson—It is an antidote in high but not in low potencies.

Dr. C. M. Selfridge—I had two cases in one family. A little girl nine years old was first taken with it; the little brother ran into the room and kissed his sister. He had been having *Belladonna*$^{200}$, three or four doses a day.

Dr. J. M. Selfridge—Hahnemann gave it in rather strong doses.

Sections 34 and 35 were read.

### Discussion.

Dr. Martin—I think this Section 34 is well illustrated in mental diseases. In chronic mania there is undoubtedly a low grade of inflammation. How many times have we seen it a fact that a person so suffering meets with an accident about the head, something which sets up an acute inflammatory condition, during which time it seems that the patient must die, and yet he gets through with that trouble and the mania is cured afterward. Nature has to put forward all her powers to cure the acute trouble.

Dr. J. M. Selfridge—This is not in accordance with the usual course of things. We often have chronic troubles following acute conditions.

Dr. Martin—These cases are really comparatively common in insane asylums. Persons who have perhaps been suffering for years, get an acute attack of some kind, and then get well altogether. I think the facts are authentic.

Dr. Chapman—I have known such cases. For instance, a lady in the East, during the menapause, thought her soul lost, etc. Her friends thought they would have to take her to an asylum. Typhoid fever attacked other members of the family, she took care of her girls, was attacked with the disease and recovered from the insanity.

Sections 36–39 were read.

### Discussion.

Dr. J. M. Selfridge—I have seen issues put on the arm in chronic coughs, setons in back of neck for headaches, etc. Moxas have been used in curvatures of the spine up and down the spine. It was done by rolling up cotton cloth like a bandage, soaked in turpentine, putting it upon the back and setting it on fire until it burned down to the flesh.

Dr. Chapman—It has been a source of wonder to me why the old-school men should not be glad to accept any new theory.

Dr. Martin—It does not seem at all strange to me that the practice of Homœopathy does not progress more rapidly because it is a fact in the history of the world that *truth and simplicity are slow to make headway.* For instance, a man comes to me suffering from some chronic trouble, and in two or three weeks under homœopathic treatment seems to be cured, and yet this will not be believed.

Dr. Augur—Since I have been practicing Homœopathy I have been astonished at the apparent lack of belief of the laity in homœopathic cures, and I have frequently said to my patients that if they had the confidence in Homœopathy that I have there would not be an allopathic physician engaged for anything. I think the fault is with the homœopaths themselves. *They are inconsistent, and do not practice what they preach.* That is why Homœopathy does not get along better. When the allopaths see that homœopaths who profess to be homœopaths do not practice what they preach, they will not believe when we do make a cure.

Dr. J. M. Selfridge—It is just as Dr. Augur says. The allopaths say that we do not do as we say. If the homœopaths would stick to their principles we would convert the allopaths.

Dr. Chapman—Our colleges do not teach as they should. They do not teach *pure* Homeopathy, but only mongrelism.

Dr. Martin—There is one fact back of it all, and it is that *in order to be a good homœopath one must work like a slave from the beginning to the end,* and unless a practitioner does that he will not be successful in the treatment of his cases. The trouble is they are too lazy, and will not give the effort to study up the proper prescription which is necessary.

All—That is what is the matter.

Dr. Wilson—When patients come to your office, and you do not do any more than give the homœopathic remedy when it is indicated, they are not satisfied, as they think that something more should be done. They seem to think that you have not paid enough attention to them.

Section 40 was read.

The President then declared the meeting adjourned, to meet again the third Friday in January at the office of Dr. J. M. Selfridge, in Oakland, when the reading of *The Organon* would be commenced at Section 41.

W. E. LEDYARD, *Secretary.*

Reported by Eleanor F. Martin, M. D.

---

